# Expanding and improving nanobody repertoires using a yeast display method: Targeting SARS-CoV-2

**DOI:** 10.1016/j.jbc.2023.102954

**Published:** 2023-01-28

**Authors:** Frederick R. Cross, Peter C. Fridy, Natalia E. Ketaren, Fred D. Mast, Song Li, J. Paul Olivier, Kresti Pecani, Brian T. Chait, John D. Aitchison, Michael P. Rout

**Affiliations:** 1 Laboratory of Cell Cycle Genetics, The Rockefeller University, New York, New York, USA; 2 Laboratory of Cellular and Structural Biology, The Rockefeller University, New York, New York, USA; 3 Center for Global Infectious Disease Research, Seattle Children's Research Institute, Seattle, USA; 4 Laboratory of Mass Spectrometry and Gaseous Ion Chemistry, The Rockefeller University, New York, USA

**Keywords:** nanobodies, monoclonal antibodies, yeast display, COVID-19, SARS-CoV-2, epitope binning, DNA shuffling, ACE2, angiotensin-converting enzyme 2, cDNA, complementary DNA, CDR, complementarity-determining region, FACS, fluorescence-activated cell sorting, FR, framework region, MAb, monoclonal antibody, MS, mass spectrometry, RBD, receptor-binding domain, S, spike, VOCs, variants of concern

## Abstract

COVID-19, caused by the coronavirus SARS-CoV-2, represents a serious worldwide health issue, with continually emerging new variants challenging current therapeutics. One promising alternate therapeutic avenue is represented by nanobodies, small single-chain antibodies derived from camelids with numerous advantageous properties and the potential to neutralize the virus. For identification and characterization of a broad spectrum of anti–SARS-CoV-2 Spike nanobodies, we further optimized a yeast display method, leveraging a previously published mass spectrometry-based method, using B-cell complementary DNA from the same immunized animals as a source of V_H_H sequences. Yeast display captured many of the sequences identified by the previous approach, as well as many additional sequences that proved to encode a large new repertoire of nanobodies with high affinities and neutralization activities against different SARS-CoV-2 variants. We evaluated DNA shuffling applied to the three complementarity-determining regions of antiviral nanobodies. The results suggested a surprising degree of modularity to complementarity-determining region function. Importantly, the yeast display approach applied to nanobody libraries from immunized animals allows parallel interrogation of a vast number of nanobodies. For example, we employed a modified yeast display to carry out massively parallel epitope binning. The current yeast display approach proved comparable in efficiency and specificity to the mass spectrometry–based approach, while requiring none of the infrastructure and expertise required for that approach, making these highly complementary approaches that together appear to comprehensively explore the paratope space. The larger repertoires produced maximize the likelihood of discovering broadly specific reagents and those that powerfully synergize in mixtures.

The COVID-19 pandemic, caused by the sarbecovirus SARS-CoV-2, has had a profound global impact the likes of which has not been seen in more than a century. The remarkably rapid development and distribution of vaccines undoubtedly saved many millions of lives (1, 2); nevertheless, at the time of writing, mortality estimates range from 10 to 20 million, with additional profound long-lasting health impacts for many survivors (3, 4). The disease appears to be transitioning to an endemic phase, thus presenting a serious worldwide health problem for the foreseeable future (5, 6) and demanding a large-scale ongoing implementation of new prophylactics and therapeutics.

Major therapeutic strategies have utilized antibodies directed against the major Spike (S) surface envelope glycoprotein of the SARS-CoV-2 virion, various fragments of which are also the immunogens for most COVID-19 vaccines. S is a homotrimer of an extensively glycosylated ∼200 kDa protein composed of two major domains: S1, which contains the host receptor-binding domain (RBD) that targets the angiotensin-converting enzyme 2 (ACE2) surface receptor on host cells, and S2, which upon host cell binding undergoes major conformational changes to enable viral – host membrane fusion, resulting in virus entry into the cytoplasm (7, 8, 9, 10, 11, 12). Thus, antibodies that target S, and particularly RBD – generated through vaccination or exogenously introduced – have the potential to block viral binding and entry into the cells of the host. Unfortunately, the continuing emergence of new SARS-CoV-2 variants of concern (VoCs; Alpha, Beta, Gamma, Delta, Omicron, and subvariants) presents a significant barrier to attaining complete control of COVID-19. These VoCs usually have many S mutations (especially in the RBD) and thus, are relatively poorly neutralized by current vaccines and antibody therapies (13, 14). For instance, monoclonal antibodies (mAbs) have proven to be an effective therapeutic strategy, though sensitive to emerging variants (15, 16). Moreover, mAbs are limited by challenges in the ease and cost of their large-scale manufacturing, distribution, and intravenous administration (17).

A promising alternative to mAbs is a particular class of single domain antibodies termed “nanobodies”. Nanobodies are “mini-antibodies,” some 1/10th the size of regular IgGs, derived from the variable domain (V_H_H) of variant heavy chain-only IgGs (HCAbs) found in camelids (*e.g.*, llamas). Each nanobody molecule is constructed of a single Ig fold, consisting of four framework regions (FRs) that intersperse and orient three complementarity-determining regions (CDRs) that form the nanobody paratope (18). These regions are similar to FRs and CDRs of conventional antibodies (19). As with conventional antibodies, CDR3 is formed by VDJ (Variable, Diversity, Joining gene segments) recombination of germline DNA; CDR1, and CDR2 come from the germline V region, and all three CDRs are then subject to somatic hypermutation with selection for improved binding affinity to antigens (19).

Nanobodies have several attractive advantages over mAbs, including: extremely fast on-rates leading to high overall affinities (20, 21, 22); characteristics of small molecules in terms of higher tissue penetration and accessibility to regions of S not accessible to the larger mAbs or occluded by glycosylation (20, 21, 23, 24) greatly enhancing their potential to synergize in combination, a profound advantage they have over often poorly-synergizing conventional antibodies (20, 25, 26). They can be readily engineered, including humanization to minimize immunogenicity; they are highly denaturation-resistant, giving them long shelf lives and making them suitable for a broader range of delivery methods (*e.g.*, *via* nebulization directly into the lungs) (27, 28); and very low cost of production in bacterial or yeast expression systems (20, 29, 30, 31, 32). Like mAbs, nanobody binding can be disrupted by mutations in VoCs. However, by producing large repertoires targeting the entirety of S, we have made many nanobodies that are resistant to the major VoCs (20). Moreover, their small size allows a high multiplicity of binding to S, which, as we have shown, allows the judicious selection of nanobody cocktails that can pack around different epitopes as well as all three copies of the same epitope; these generate a tremendous synergistic effect that can also be highly VoC resistant, as the virus must now evolve multiple simultaneous mutations to evade such cocktails (20).

To produce such valuable nanobody repertoires, we have previously employed a mass spectrometry (MS)-based approach (20, 22) in which we immunized llamas with S constructs, taking advantage of powerful natural affinity maturation processes *in vivo* (33), and then performed high-throughput DNA sequencing of V_H_H libraries PCR-amplified from marrow lymphocyte complementary DNA (cDNA) from the immunized llamas in combination with MS identification of high-affinity V_H_H regions derived from the serum of the same animal. Computational matching of MS-sequenced peptides to V_H_H cDNA sequences allowed high-confidence identification of sequences encoding high-affinity nanobodies. Genes encoding nanobodies were synthesized and expressed in bacteria, and nanobodies were purified and characterized for their specificity and affinity. However, these V_H_H cDNA libraries also represent a resource that can be tapped for an orthogonal approach for nanobody production, employing display screening methods instead (34, 35, 36, 37). Potentially, this approach could discover additional nanobodies to enrich our repertoires and also serve as a convenient platform to explore the specificity and VoC sensitivity of a large number of nanobodies in parallel. Recently, a robust and efficient yeast display method was designed and validated specifically for screening nanobodies, in particular against S (34, 38). In that work, a synthetic nanobody library was employed. Here, we chose to evaluate instead the cDNA library made from immunized llamas and already tested and mined by the MS-based approach (20), which we transferred into the nanobody display vector (34). We tuned the display approach and selected large repertoires of nanobodies that were specific for different domains of S and contained members that displayed high affinity and resistance to VoCs and also employed DNA shuffling of the CDRs to generate variants with novel VoC specificities. We show that the yeast display method, either on its own or in parallel with the MS method, can generate large nanobody repertoires with high potential as therapeutics, in this case against COVID-19.

## Results and discussion

### Optimization of yeast display screening

**Figure 1:**
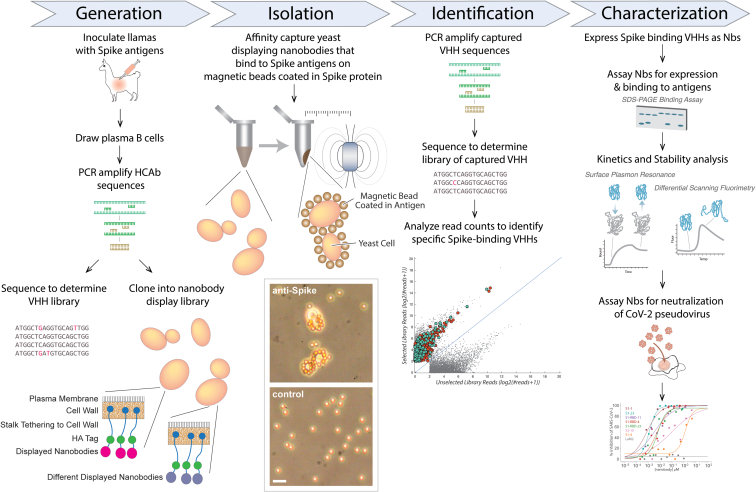
**Design of yeast-based nanobody screen for anti-SARS-CoV-2 spike domains.** Schematic of our yeast display based strategy for generating, identifying, and characterizing large, diverse repertoires of nanobodies that bind the spike protein of SARS-CoV-2. The highest quality nanobodies were assayed for their ability to neutralize SARS-CoV-2 pseudovirus. Figure adapted from (20); diagram of yeast display construct (*bottom left*) adapted from (34). Boxed inset shows 2.8 μm magnetic beads conjugated with S1 protein from SARS-CoV-2 Spike, after a binding reaction with yeast displaying an anti-S1 nanobody (*top*) or a nonspecific control (*bottom*), after nonbinding yeast were washed away. The scale bar represents 10 μm.

**Figure 2:**
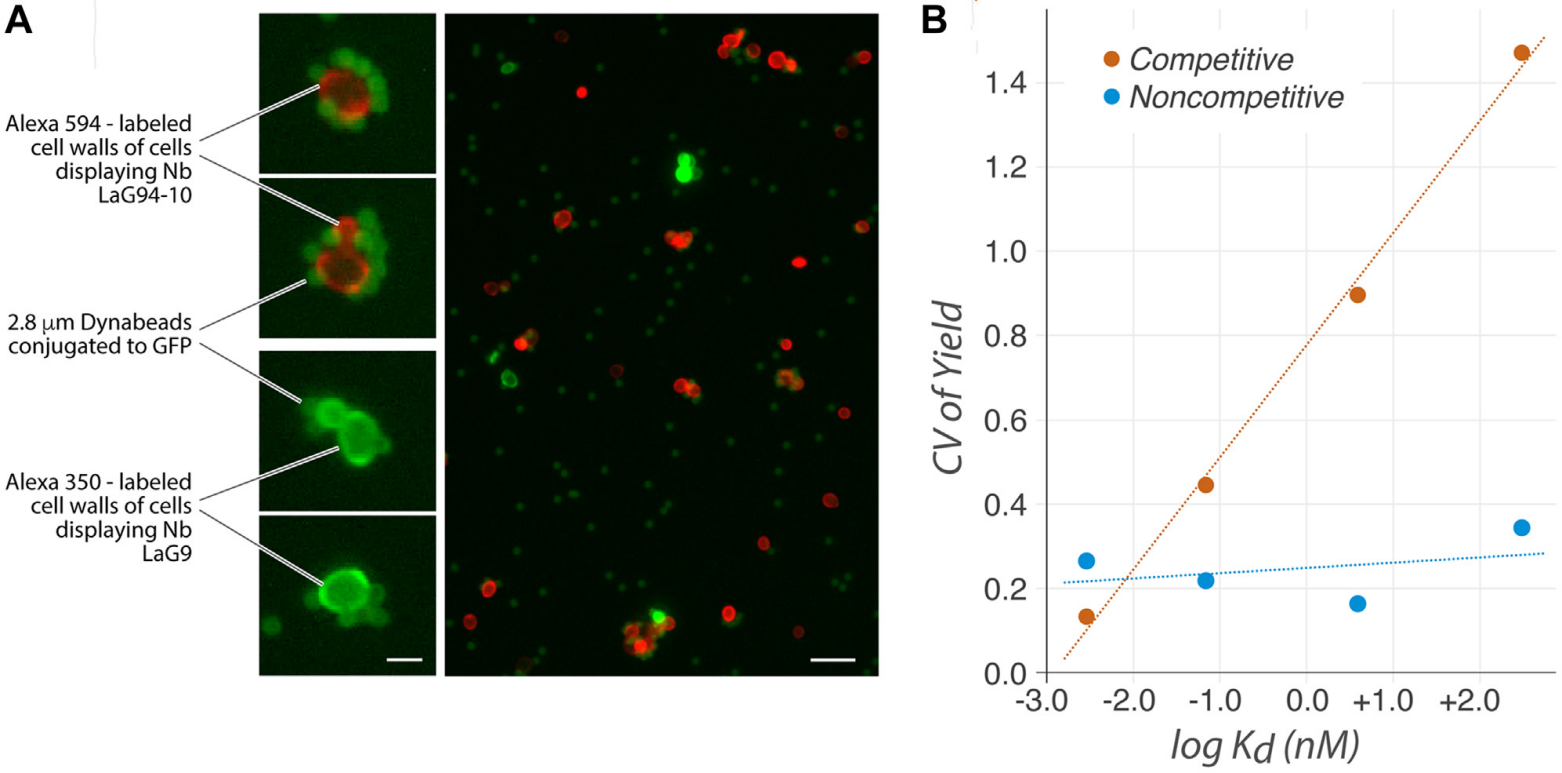
**Characterization of competition effects between the yeast displaying nanobodies of different defined affinities.***A*, fluorescence microscopy of a yeast competition assay. Here, yeast displaying either LaG94 to 10 (Kd = 2.9 PM) or LaG9 (Kd = 3.9 nM) anti-GFP nanobodies were live surface-labeled with either Alexa594 (*red*) or Alexa350 (*green*) (40). These were mixed together in equal proportions (50:50:0, “competitive” conditions; see text) with GFP-conjugated 2.8 μm magnetic beads (*small green spheres*), to which they bound (*left panels*, the scale bar represents 5 μm; see also Fig. 1). *Right panel* shows a low magnification field of the assay after several rounds of harvesting and washes, showing in this case the enrichment of the LaG94 to 10 displaying yeast; the scale bar represents 20 μm. *B*, plot of the coefficient of variation (CV) of yield for each yeast strain across the experiments in *B* against the Kd of their displayed nanobodies. As seen in the plot, the yields of yeast bearing low-affinity nanobodies were highly variable, specifically under competitive conditions. The yield of the highest-affinity Nb-bearing yeast, in contrast, were almost invariant because they always won the competition. Under noncompetitive conditions, this differential was lost.

**Fig S1:**
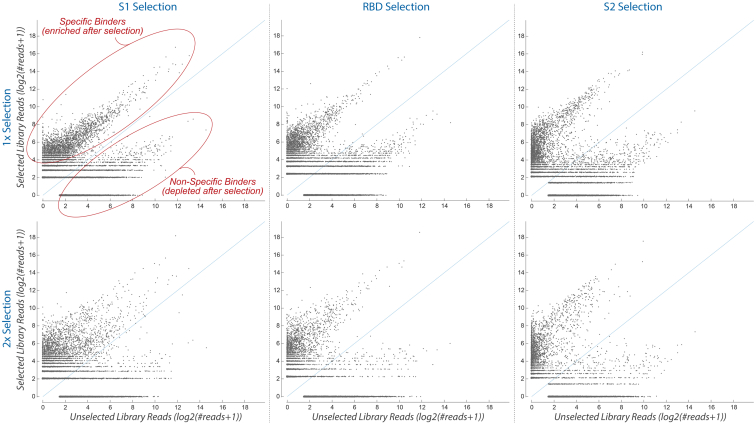
Binding of nanobodies expressed in yeast to the different recombinant S1, RBD, and S2 domains of SARS-Cov2 spike protein (labeled S1, RBD, and S2 selection, respectively) (20) conjugated to Dynabeads. Binding and washing were as described in Methods. Nanobody sequences were amplified and sequenced as in Fig. 3. The CDR regions were extracted from each sequence and concatenated into a “CDR string”. The read counts for each unique CDR string in the unselected library and the selected libraries were compared and, after standardization to reads per million nanobody sequences, plotted (*gray points*). Because of the wide spread of values, we plot the log2 of these values +1 (the addition of 1 allows plotting of zero reads as the value 0). For all three antigens, we observe two lines of points, one above the x = y line and one below. Both lines trend upwards with increasing read count in the unselected library. We take the upper line to represent specific binding (enriched; labeled with *red oval* as “specific binders”), and the lower line to represent nonspecific binding (depleted; labeled “nonspecific binders”); the *blue line* (x = y) thus represents neither enrichment nor depletion. Both lines increase with a slope of 1, probably simply because there are more yeast bearing that sequence due to differential abundance in the llama. The vertical displacement of the specific from the nonspecific line represents the overall enrichment of specific over nonspecific binding in our conditions, approximately 1000-fold. This is in agreement with the differential binding we achieved comparing yeast with a control surface nanobody to yeast with a high-affinity anti-GFP nanobody to Dynabeads bearing GFP (Fig. 2). 1 × selection: one round of binding. 2 ×: selection: yeast from the 1× selection were grown out, expression of the nanobody fusion reinduced, and the yeast were bound to the same antigen (see Methods). The main effect of this was to reduce the population of the lower (nonspecific) line

We show our general pipeline design in Figure 1. We used a well-characterized nanobody display vector (34), which gave efficient and selective binding of specific nanobodies to diverse targets in our hands (GFP, S1, RBD, and S2 regions of S). Instead of published approaches such as rounds of fluorescence-activated cell sorting (FACS) (39) or Miltenyi magnetic bead and biotin-based purification followed by FACS (34), we employed Dynabeads coupled to antigen for all selection steps (Fig. 1, inset). For most of initial experiments, we generated a set of four anti-GFP nanobodies in the display vector (22) with a broad range of known affinities, and then tested for the binding of yeast expressing these to GFP-conjugated Dynabeads, as well as an irrelevant nonspecific nanobody control. We also cloned three previously characterized anti-S nanobodies (20) into the yeast display vector to test their binding to various S domain-conjugated Dynabeads. We used all these well-characterized nanobodies as benchmarks to optimize conditions in terms of simplicity, specificity, and yield, trying for an optimal combination of high binding of specific nanobody-expressing yeast and low binding of control yeast carrying just the vector. We tested a range of binding buffers and conditions to optimize specific binding to these beads, taking advantage of the extreme robustness of yeast to even harsh binding conditions, including high salt as well as acidic and detergent washes. We found that a straightforward buffer, related to the ones used for immunofluorescence microscopy and immunoblotting, gave excellent signal-to-background with these benchmarks (see Methods). Often, for specific binding, multiple beads were attached to each yeast cell, whereas no beads would be seen bound in the nonspecific control (Figs. 1 and 2*A*). Magnetic isolation of the Dynabead-binding yeast was found to give the highest yields while maintaining efficient removal of nonspecific yeast when the magnet was placed at a distance from the yeast washing suspension such that it took several minutes to fully harvest the beads; a closer placement of the magnet led to loss of cells during the repeated washes. Care was taken on each wash step so that the beads were fully resuspended with minimal displacement of specifically-attached yeast from them. We found that 4 to 5 wash steps were sufficient to achieve a 100- to 1000-fold enrichment of specific binders over the control in a single round of isolation, which was in turn sufficient for clear identification of specific binders by comparison of sequence counts before and after binding (Fig. S1).

### Determination of the roles of nanobody affinity and competition during screening

Competition has the potential to dramatically limit the repertoire, so we sought conditions where we could isolate high-affinity nanobodies but not at the expense of competition among yeast – an effect that could be exacerbated by avidity effects. Therefore, we used our model nanobody display strains to determine the avidity effects of the many nanobodies displayed per yeast cell the and competition between yeast cells displaying nanobodies of differing affinities for the same antigen. In order to distinguish the cells of strains displaying nanobodies of differing affinities, we covalently stained cell walls with vital fluorescent dyes (40) (Fig. 2*A*). To examine the effects of competition, we mixed equal cell numbers of two strains of yeast, each uniquely dyed and expressing one of two different anti-GFP nanobodies of differing affinities (22), and also unlabeled control cells expressing an irrelevant nanobody. These three cell populations were in ratios of 50:50:0 or 1:1:98 respectively, all at the same final cell density and volume; the amount of GFP-Dynabeads was also held constant. Initial experiments suggested that in the 50:50:0 condition, beads might be limiting, since we observed depletion of free beads not bound to yeast in the final harvest (see Fig. 1 for an example of bead depletion). In contrast, at the 1:1:98 ratios, there appeared to be abundant beads remaining after binding and washes. Therefore, we call the experiment carried out at 50:50:0 “competitive” and the 1:1:98 “noncompetitive”.

To assess the effect of competition as a function of binding affinity, we quantified the variability of yield across all possible pairwise binding experiments (Fig. 2*B*). The yield of yeast displaying low-affinity nanobodies was highly variable, because when competing with yeast displaying successively higher affinity nanobodies, the yield of the former was increasingly depleted. This effect was far stronger in competitive versus noncompetitive conditions. In contrast, yeast expressing high-affinity nanobodies displayed little variation in yield even under competitive conditions, because they “won” the competition. These results clearly demonstrate that the yeast display system is sensitive to the monomer-binding affinity of the displayed nanobodies. We find this result surprising due to avidity considerations, but the effect seems quite clear and was further validated in testing anti-S nanobodies of known affinities (see below). This effect would become exacerbated under the increasingly competitive conditions that will arise through repeated rounds of panning characteristic of most display methods, leading to isolation of the most competitive clones at the expense of other less competitive but still potentially valuable high-affinity clones. Thus, we instead sought to take advantage of (i) starting with a hyper-immune animal and (ii) the high signal-to-background of our optimized panning conditions to limit our screens to just two rounds of panning, which we found to be sufficient under these conditions to identify strong positive clones that outcompete nonspecific or low-affinity clones, but not at the expense of outcompeting other valid high-affinity clones.

### Examination of the sequence diversity of the yeast display libraries

**Figure 3:**
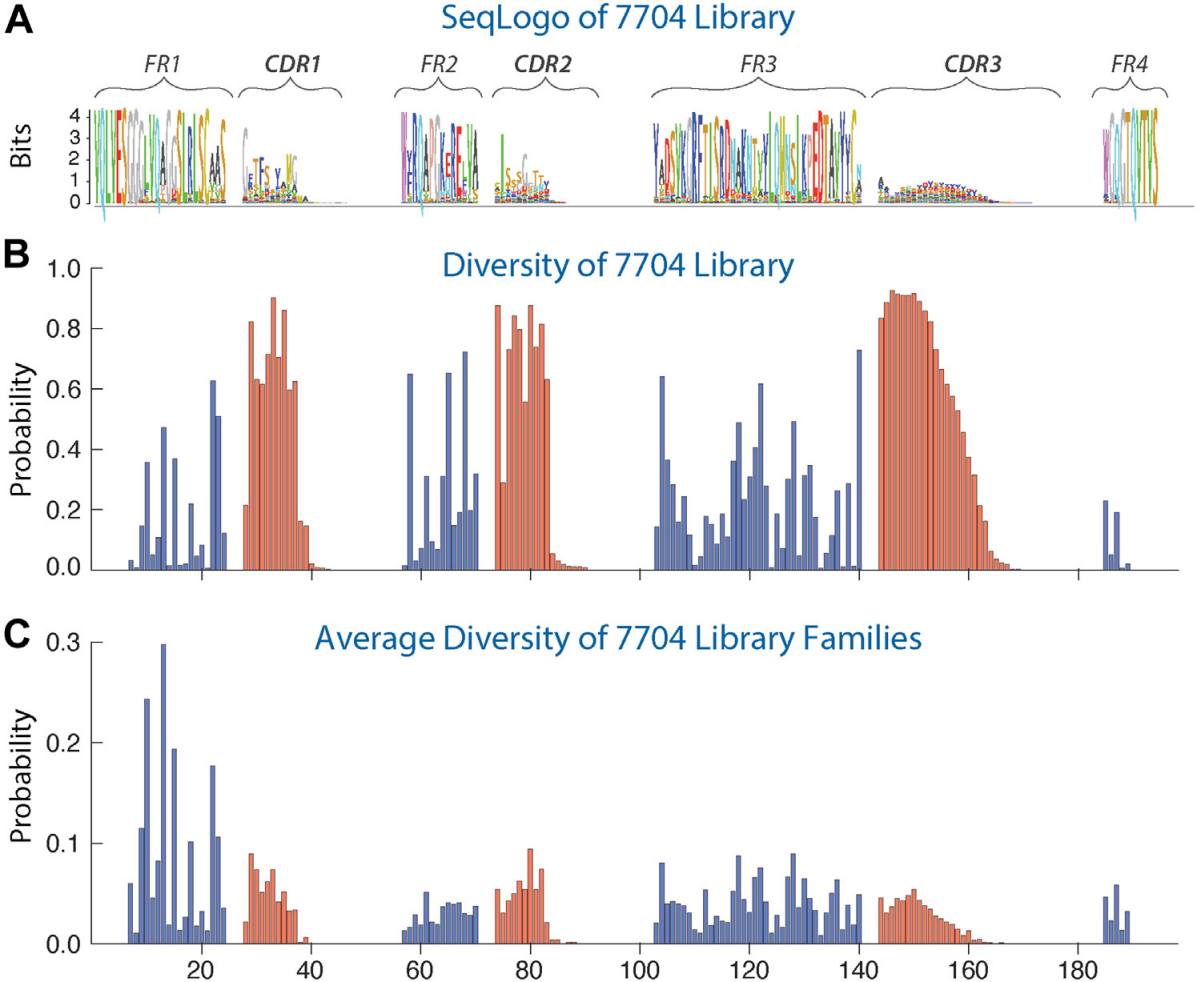
**Sequence diversity of nanobody libraries.** Nanobody sequences from the unselected yeast library (from llama 7704; ∼1.5 × 10^7^ independent clones) were amplified and sequenced with Illumina Miseq and processed to minimize sequence errors, as described in Methods, yielding 1.2 × 10^6^ distinct nanobody sequences. Framework regions (FRs) and CDRs were determined in each encoded nanobody based on the alignment of (41). Unique sequences were determined and aligned by left justification of each region. *A*, a seqlogo (44, 59) was generated (MATLAB seqlogo command). High variability in the three CDR regions is evident. *B*, plot of proportion of nonconsensus residues per residue across the library. At each position the sequence diversity was calculated (defined as the probability that two randomly chosen sequences are identical at the position (60). High variability in CDRs (*red bars*) is again observed, as well as lower but significant variation in the FRs (*green bars*). *C*, plot of sequence diversity per residue across the library (60). The library was sorted into CDR3 “families”: groups of unique sequences (minimum family size 100) differing by no more than 20% in any of the seven regions (this criterion extracted about 15% of the unique sequences into 370 CDR3 families). Because this criterion results in high similarity of CDRs within a CDR3 family, it is very likely that the sequences in each CDR3 family derive from a unique VDJ (Variable, Diversity, Joining gene segments) recombination with subsequent somatic hypermutation. For each CDR3 family the sequence diversity was calculated, and the aggregate average diversity graphed as in *B*. As expected, the diversity within CDR3 families was significantly reduced compared to the whole library (note difference in y-axis scale), and the differential between FRs and CDRs largely lost.

We used gap-repair in yeast to clone into the yeast display vector B-cell V_H_H cDNA from two llamas immunized with S proteins, 5094 (Marley) and 7704 (Rocky) (20). We obtained libraries of respectively ∼5 × 10^6^ and ∼1.5 × 10^7^ independent clones. We carried out next-generation sequencing on the inserts amplified from the transformed yeast. We observed highly diverse sequences, especially in the known hypervariable regions CDR1,2,3, and also considerable though lesser variation in the framework regions (Fig. 3, *A* and *B*). We noted that the library contained many “families” of closely related but distinct sequences. Variability within families was reduced compared to variability in the library overall but was still substantial even in the framework regions (Fig. 3*C*). Positions of variation in framework regions within families were similar to the variable positions in the overall library. We believe this is likely due to these variable positions occupying loop or surface positions far from the antigen binding site in the nanobody structure, since this has been noted previously for positions of nanobody variation (41, 42). This, in turn, suggests that diversity within these sequence families was generated under selection in the llama, implying that this variation is largely due to somatic hypermutation after “founding” of the family by V-D-J recombination (see Introduction) rather than artifactually introduced during cloning and sequencing. The multiple related sequences in these families effectively provide a large number of biological replicates for binding experiments, as shown below.

### Validation of the yeast display method with biochemically identified nanobodies

**Fig S2:**
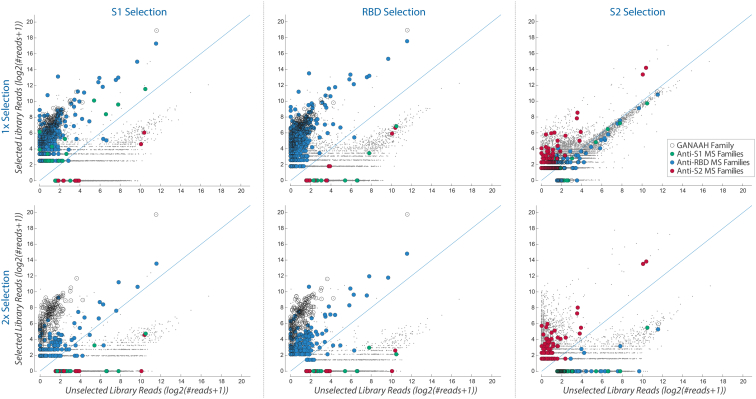
Validation of yeast display method with MS-positives from another llama. In (20), we searched for nanobodies in two llamas, 7704 (“Rocky”) and 5094 (“Marley"). High-affinity binders were recovered from llama 5094, though apparently fewer than from 7704, reflecting the results of (20). We carried out the same assay for these nanobodies and plotted the results as in Fig. 4*A*, with the exception that to get sufficient representation, we accepted CDR strings with up to 20% sequence variation in each CDR; each different color of the dots plotted represents a member of a family of a given mass spectrometry-positive nanobody. We observed the same high specificity and recovery in yeast display for the 5094 clones as for the 7704 clones. Note that in contrast to 7704, 2× binding was required for a clean result in some cases; this is likely due to the overall lower anti-spike nanobody content in 5094 noted previously (20), which increases the level of nonspecific binding in the 1×.

We screened the yeast display libraries from the two S-immunized llamas using the protocol optimized with the control strains (above; see Methods). Dynabeads coupled to S1, RBD, or S2 each bound to approximately 1% of the clones in the yeast library. To evaluate this binding reaction, we amplified and sequenced the selected clones and compared the recovery of sequences to their representation in the unselected library shown in Figure 3. To simplify the analysis as well as to focus on the major paratope (*i.e.*, epitope-binding) region of each nanobody, we reduced each sequence to a representation consisting solely of its three CDR regions (CDRs 1,2,3) (we refer to this as the “CDR string”). We plotted the log2 of read count for each CDR string in the unselected library vs. the log2 of read count in the selected libraries (in all cases after correction to reads per million total sequenced). We selected over two rounds with the viral antigens coupled to Dynabeads (“1×” and “2×”). Fig. S1 shows the behavior of the entire library. We observe two broad lines of plotted points, one showing strong enrichment (ratio of selected to unselected much greater than 1, shown by the blue line) and one showing strong depletion (below the blue line). Both broad lines have slopes of ∼1, indicating a first classification of sequences into specific and nonspecific binders, as indicated in red in Fig. S1. The rising slope in both cases is due simply to more recovery of sequences that are more abundant in the initial library (showing why it is essential to have sequences for the initial as well as the selected libraries). Similar plots were obtained for all three viral antigens (Fig. S1) and both libraries (Fig. S2).

**Figure 4:**
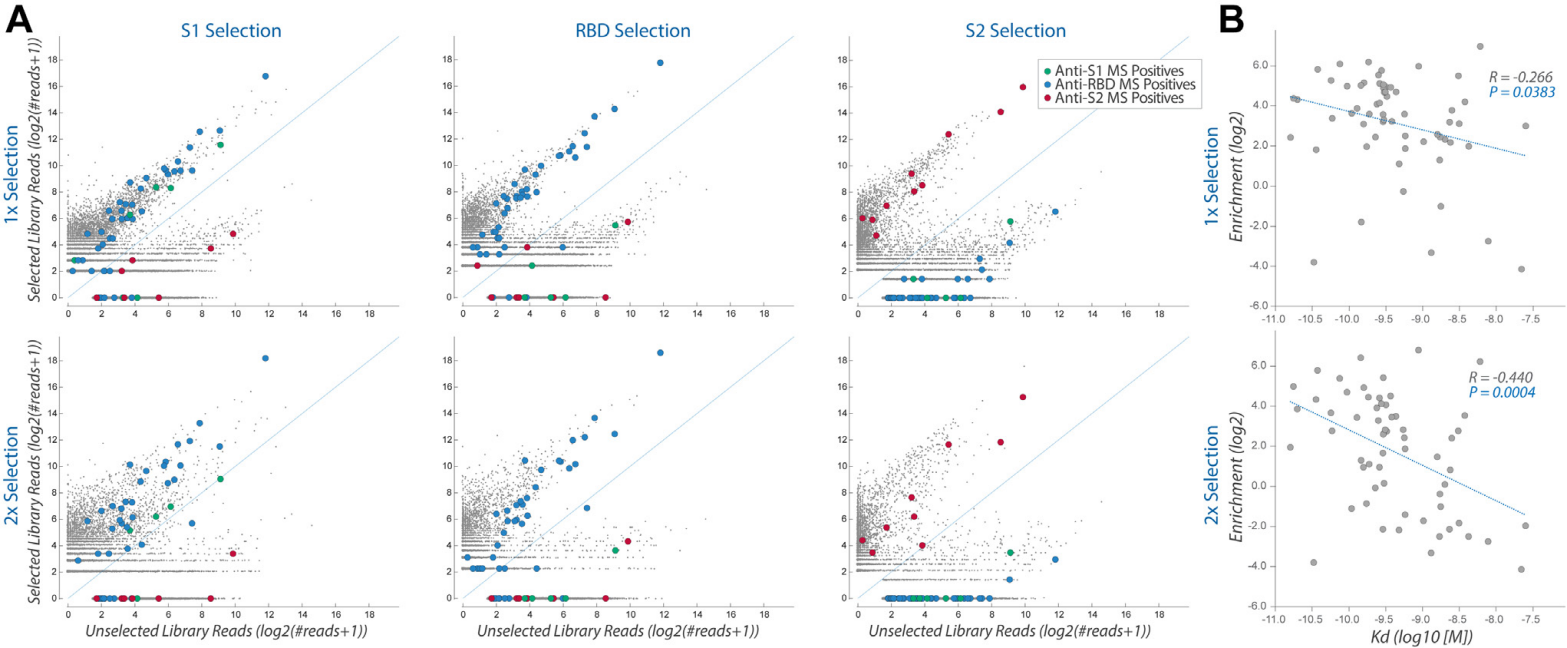
**Validation of the yeast display method with biochemically identified nanobodies.***A*, behavior of the yeast clones carrying CDRs matching the mass spectrometry positive nanobodies (*i.e.*, “MS positives”) in the spike domain affinity selection assay. The read counts in the unselected and selected libraries for all these sequences are plotted on top of the plot of the overall results from the entire llama 7704 nanobody cDNA display library (*gray points*), as plots of the log2 of these values + 1, as shown in Fig. S1. 1× selection: one round of binding. 2×: selection: yeast from the 1× selection were grown out, expression of the nanobody fusion reinduced, and the yeast were bound to the same antigen (see Results, Methods); screens were for binding to either the S1 domain (S1 selection), RBD domain (RBD selection), or S2 domain (S2 selection) of spike. In *green* are nanobodies that were shown previously to bind to S1 but not RBD; in *blue* are those that bound to both RBD and S1; in *red* are binders to S2. Note that all these nanobodies bound with precisely the specificity based on prior biochemical characterization (20) with the exception of a small number that failed to bind in any of the selections. These “MS positives” generally bind well in both the 1× and 2× selections. These results support the specificity and comprehensiveness of the yeast display procedure. *B*, correlation of yeast display binding with the affinity of their displayed nanobodies, for the MS-positives plotted in part *A*. Plot of enrichment (log2(bound)-log2(unselected) versus log10(nanobody Kd). Top: 1× selection. Bottom: 2× selection. Pearson R values and associated *p* values by *t* test are shown. The figure shows a noisy but significant correlation between binding affinity and enrichment by yeast display binding, especially for the more highly competitive 2 × binding assay (see text). cDNA, complementary DNA; MS, mass spectrometry; RBD, receptor-binding domain.

**Fig S3:**
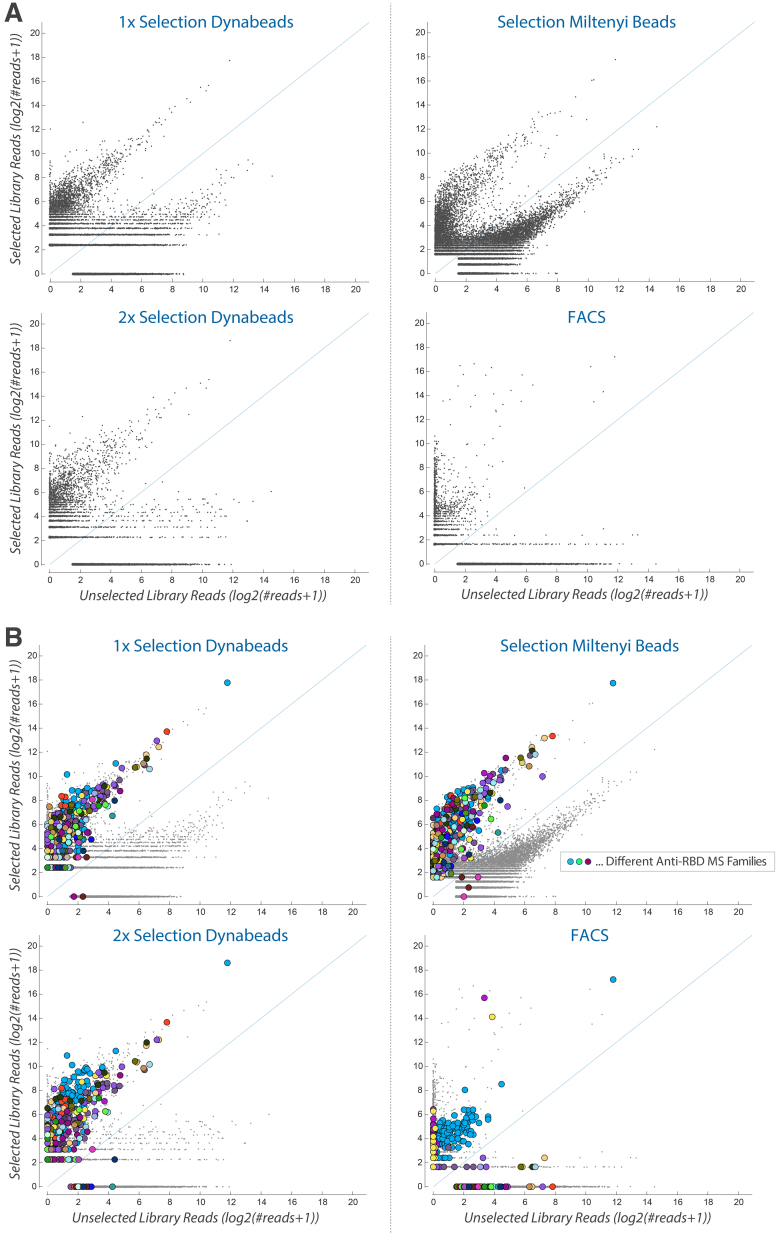
Comparison of different methods for screening the display libraries. The entire llama 7704 nanobody cDNA display library was screened against the RBD domain of spike and plotted as in Fig. S1 (*gray points*). Two methods of screening were employed. The first was as described in Fig. 1 and Methods, using two rounds of Dynabead selection. The second was following a published procedure, with the first selection utilizing Miltenyi magnetic beads and biotin purification, which was followed by a second selection utilizing a FACS sorting protocol with Alexa-labeled RBD (34) (Methods). A. All sequences recovered are plotted as in Fig. S1. B. Families of MS-positive sequences are plotted on top of the overall graph, as in Fig. S1.

Previously, we identified 374 unique CDR3s by using a biochemical and mass spectrometry-based method and from them cloned and expressed 116 high-affinity anti-S nanobodies (20), here termed “MS positives”. Since the yeast display libraries here employed the same nanobody cDNA used in that study, we used the biochemically characterized nanobodies as fiduciary markers to analyze their behavior when displayed by yeast. Importantly, these nanobodies bound with the expected specificity when expressed in yeast. Similar results were obtained with both animals (Figs. 4*A* and S2). For 5094, to increase representation due to lower overall anti-S nanobody levels in this animal as described previously (20), we also plotted close relatives (no more than 20% sequence divergence in any CDR) of the MS positives. The screen clearly distinguished specifically binding and nonbinding clones; for example, biochemically defined S2-specific nanobodies bound to S2 beads but not S1 or RBD beads when expressed in yeast, and vice versa. We plotted enrichment of these MS-positive sequences after both one and two rounds of selection against their measured K_D_s (20). While the relationship was noisy, a statistically significant negative slope was observed (Fig. 4*B*), especially for two rounds of selection (likely to be more competitive conditions based on our analysis of anti-GFPs above), once again confirming the competitive and affinity-sensitive nature of the screen. Collectively, these results validate the screen’s ability to identify large numbers of *bona fide* high-affinity S-binding nanobodies, as well as revealing two other key behaviors. First, members within a related family behave similarly in terms of relative enrichment and specificity during panning (*e.g.*, Figs. S2 and S3; see also following figures). Second, some families came to dominate the final panned populations, particularly after two rounds of selection; for example, clones with a CDR3 containing the sequence string “GANAAH” made up more than 90% of the recovered sequences from 5094 with RBD- or S1-Dynabeads (Fig. S2). However, overall diversity among the positive families was still preserved despite the distortion of representation.

We were also interested to employ this same benchmark to evaluate previously described panning methods (34, 38), in which after a first round of magnetic bead purification, subsequent panning rounds utilize flow cytometry after binding of fluorescent RBD (Fig. S3). We found the first step of the previously described method, with magnetic bead-based enrichment of binders, gave similar results to our procedure. In our hands, the second flow cytometry-based step very effectively eliminated nonspecific binders; however, we also noted that the broad representation observed with our method was lost, and the recovered clones were strongly dominated by a single sequence (with identical CDRs to the MS-positive S1-RBD-38 (20)). The stringent removal of nonspecific binders is likely critical for screening naive libraries as in (34, 38), but we find that it is not required in our context, starting with V_H_H cDNA from hyperimmunized llamas, where our method clearly preserves the high diversity present in the original cDNA.

### Screening for new families of anti-S nanobodies

**Figure 5:**
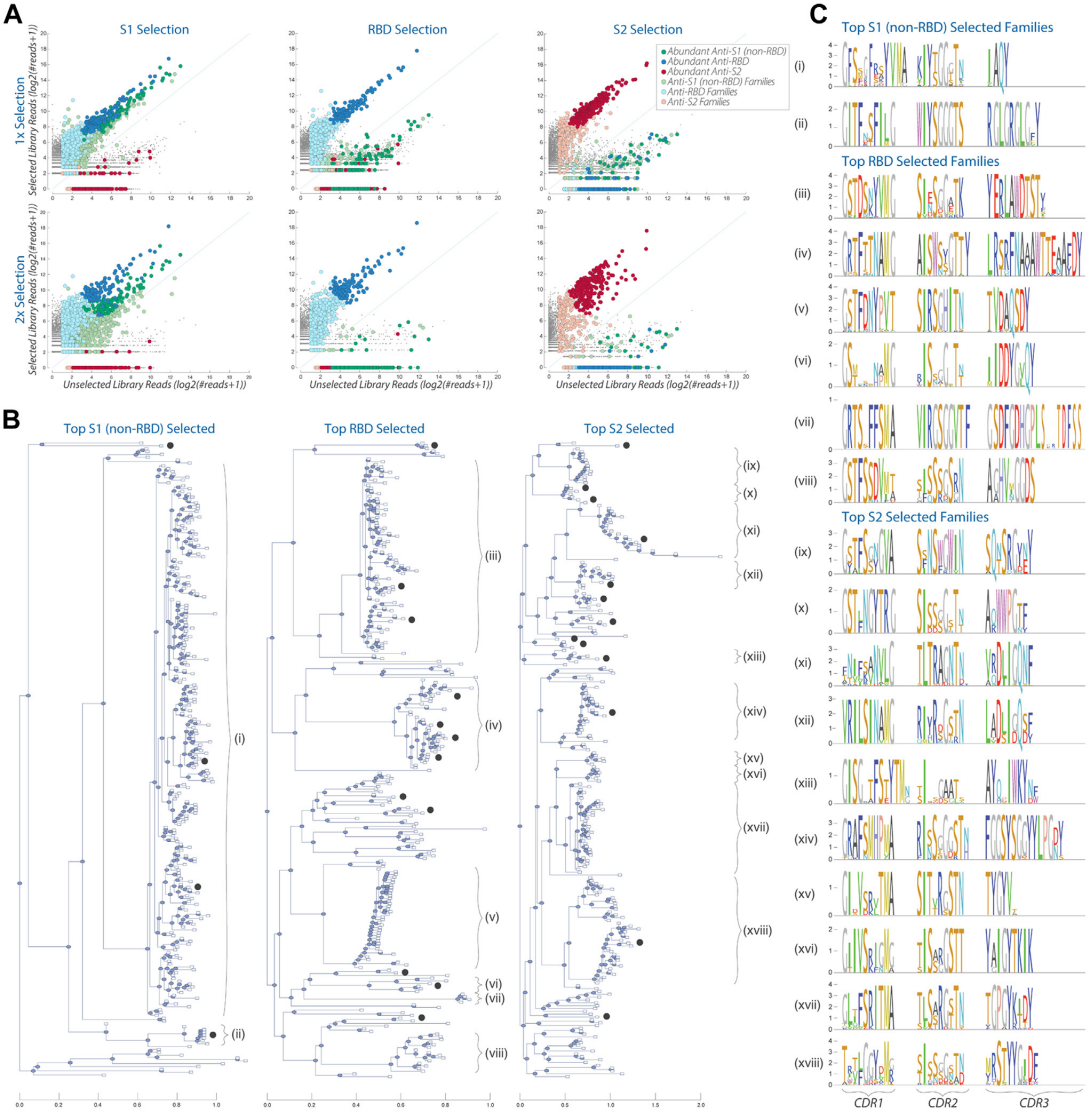
**Screening for new families of anti-spike nanobodies.***A*, the read counts in the unselected and 1× and 2× selected libraries screened against either the S1, RBD, or S2 domains of spike from the entire llama 7704 nanobody cDNA display library (*gray points*) are plotted, as the log2 of these values + 1, as shown in Figure 4. Sequences displaying different specificities were identified and selected from the graphical display as in Figure 4, except here nanobodies specific for only the RBD subdomain of S1 were separately labeled from those that recognized the non-RBD portions of S1. Selection was by a differential polygon function allowing multiple criteria. For example, sequences in an enriched polygon for S1 but not for RBD defines nanobodies binding to the non-RBD subdomain of S1 (also see Introduction). *Green*: S1 non-RBD; *blue*: RBD; *red*: S2. The *darker colors* show the initial CDR families taken from the ∼200 highest read counts sets; the *lighter colors* represent an extension of these CDR families to a broader set allowing up to 20% changes in any of the three CDR sequences, and above a minimum read count. Note that all these CDR families are bound with consistent specificity, indicating that in general, sequence variations within the families (see Fig. 3) do not strongly affect function. *B*, to evaluate sequence diversity within these functional classes, we employed the MATLAB command “phytree” to construct a neighbor-joining tree for the ∼200 abundant CDR sequences in each class (*i.e.*, using the sequences identified by the *dark green*, *blue* or *red points* in panel *A*). A wide diversity of sequences was observed, which sorted out into a more limited set of CDR families of related sequences. Families with generally larger representation are labeled with Roman numerals; *black dots* indicate sequences that were expressed and characterized as recombinant monomers (see below); these were chosen from the tree to sample broadly across the sequence space, while minimizing repetitive sampling of families with clones already characterized from the mass-spectrometrically identified nanobodies (20). *C*, sequence conservation within the CDR families indicated in (*B*) was evaluated using the MATLAB seqlogo command (44) applied to the CDR strings of the selected CDR families; shown are SeqLogos of (*left* to *right* in each SeqLogo) CDR1, CDR2, and CDR3. cDNA, complementary DNA; RBD, receptor-binding domain.

We next analyzed the screens described previously and in Figure 4 to discover novel nanobodies not previously identified by the MS method, focusing mainly on the more diverse repertoire of animal 7704 (20, 43). We selected for analysis, using a polygon function (see Methods), the ∼200 most abundant and highly enriched clones from the first round of antigen selection specifically for that given antigen, and the CDR families for those ∼200 clones were also selected (dark and light colors, respectively; Figure 5*A*, upper row). These were found to comprise the most highly enriched clones from the second round of antigen selection and preserved their antigen specificities (Fig. 5*A*, lower row).

We examined the sequence diversity and content of these positive classes by examination of neighbor-joining trees (Fig. 5*B*). While many of the sequences were similar or identical to the previously described nanobodies from this llama identified by the MS-based approach (20), we also observed many new sequences. We used CDR3 as our benchmark for defining a given family, because its generation by VDJ recombination is the unique clonal event that founded a large cluster of related sequences by somatic hypermutation. The estimates for the number of families that recognize each S domain vary depending on the sequence parameters used to define each family, the antigen in question, the cutoff for enrichment used during a panning round, the cutoff for minimum read count (to exclude spurious sequencing/PCR errors), and the minimum size of a family. We used similarities within CDR3 to define families, with each family being defined as being made of members related to each other within a certain value or less of sequence identity. For example, for animal 7704 with the more inclusive parameters of 70% or more CDR3 sequence identity and a minimum enrichment of twofold, we found 259 S2-specific families, 90 RBD-specific families also recognizing S1, and 60 S1 (non-RBD) families. Interestingly, there are a further 106 families, mostly low read count, that recognize RBD but not S1, presumably antigens buried in the full S protein. By any count, however, it is clear that a very large number of anti-S families – often over one hundred per domain – can be identified by this approach.

We constructed a neighbor joining tree, though not a “phylogeny” tree in the strictest sense, but the nodes nevertheless correspond to the sequence families discussed previously (see also (20)). Major representative families are denoted in Figure 5*B* with a roman numeral, and Seqlogos (44) were constructed for these families (Fig. 5*C*). It can be seen that the CDR1,2,3 sequences are very diverse between families but largely contain only minor variations within families. Moreover, a given family shows absolute antigen specificity, such that, for example, an RBD-specific family is not found enriching in an S2 screen. The S1-non-RBD clones are dominated by members of one family, defined by their “IAQY” consensus CDR3 sequence (Fig. 5, *B* and *C*; family (i)). This family was missed in the MS-based approach (20), possibly because the CDR3 was too small for reliable peptide identification. However, numerous other new S1-specific families are present, for example, the “RGLGRGLGFY” CDR3 consensus sequence (Fig. 5*B*, family (ii)). By contrast, and in agreement with its antigenic nature (12, 20, 45, 46), the RBD domain isolated a large and diverse set of families. One of the largest families (Fig. 5*B*, family (v)) contains the consensus CDR3 “TVDAQSDY”, which is also found in the mass spectrometrically identified nanobody S1-RBD-38. Two large families identified here contain divergent relatives among the MS-identified nanobodies (20), the “LRSRFNAAAWTTEAAFDY” (previous MS-identified S1-RBD-6 and S1-RBD-31) and “YERLAWDTSTY” families (previous MS-identified S1-RBD-35), the remaining four indicated families being completely novel. The S2-specific clones were also very diverse and not dominated by any single family (Fig. 5, *B* and *C*), with limited overlap with the mass spectrometrically identified clones (20). Collectively, the screening method identified a large number of new clones recognizing different S domains. It then became important to determine if these new clones had high-affinity binding and strong antiviral activity when expressed as monomers.

### Testing the library against the major VoCs Delta and Omicron

**Figure 6:**
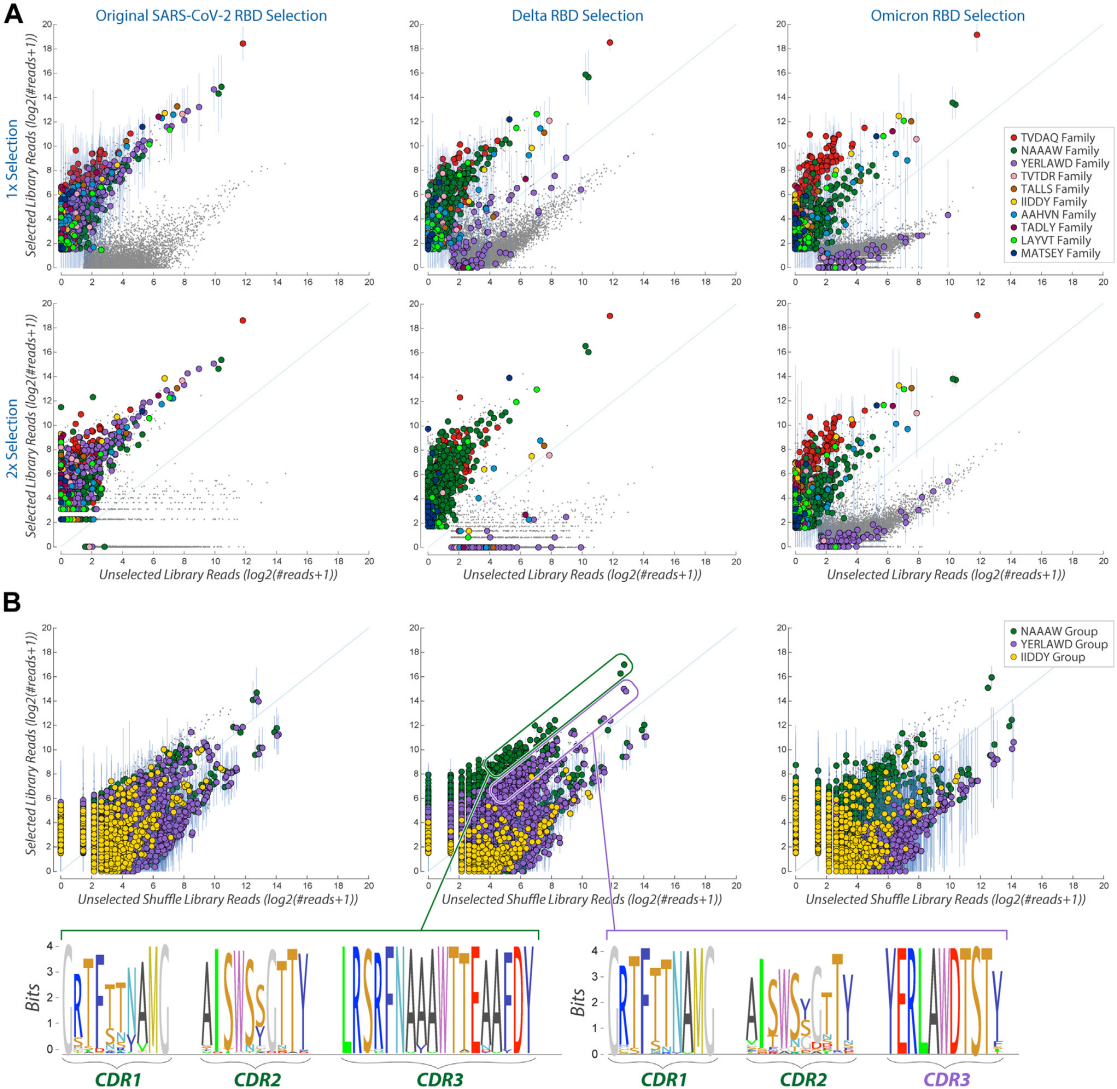
**Testing the standard and shuffled libraries against RBD variants Delta and Omicron.***A*, testing the standard library against RBD variants Delta and Omicron. Dynabeads conjugated with RBD from the original SARS-CoV-2 and from the Delta and Omicron variants were employed for affinity purification of yeast display clones from the llama 7704 library. Two rounds of selection were carried out, as in Figures 4 and 5. A clear overall reduction in binding was observed, though many clones still bound well to both variants. Plotted on top of the overall library result (*gray points*) are sequence families illustrating some of the main patterns of response to the variants are indicated as colored dots, with each color corresponding to the indicated family, defined here by their core CDR3 amino acid sequence. These families contain considerable sequence diversity within them (Fig. 5), and represent much larger numbers of many other unrelated clones (often with lower representation in the library) that exhibit similar behaviors. *B*, testing a shuffled anti-RBD library for CDR autonomy and rescued activity in recombinants. (*Top*). Binding of the shuffled library to RBD. One round of purification was carried out, followed by sequencing. Results were analyzed based on the fate of CDR3 “groups” (where within a group, a given CDR3 bound to a high diversity of CDR1,2 recombinants). Different behaviors were observed; three are illustrated with colored dots (same color scheme as Fig. 7). The “NAAAW” group (*green*) bound well to original and variant RBDs, essentially independent of the recombinant CDRs 1 and 2 it was attached to, suggesting strong “CDR3 dominance” of the “NAAAW” CDR3. Similarly, the “IIDDY” group exhibited essentially similar behavior when shuffled as when combined with its native CDR1,2 (Fig. 7): strong binding to original and Omicron, but clearly weaker binding to Delta. The “YERLAWD” recombinant group bound comparably to all variants, in contrast to its behavior with its native CDRs 1 and 2, which rendered it unable to bind Delta or Omicron. (*Bottom*). Effectiveness of the shuffle is shown by extracting all sequences bearing a specific CDR3 and examining sequence diversity of CDRs 1 and 2. Sequence logos show that highly diverse CDRs 1 and 2 are observed joined to each of three different CDR3’s, and the pattern of CDR1,2 diversity attached to the CDR3’s was essentially the same. RBD, receptor-binding domain.

**Figure 7:**
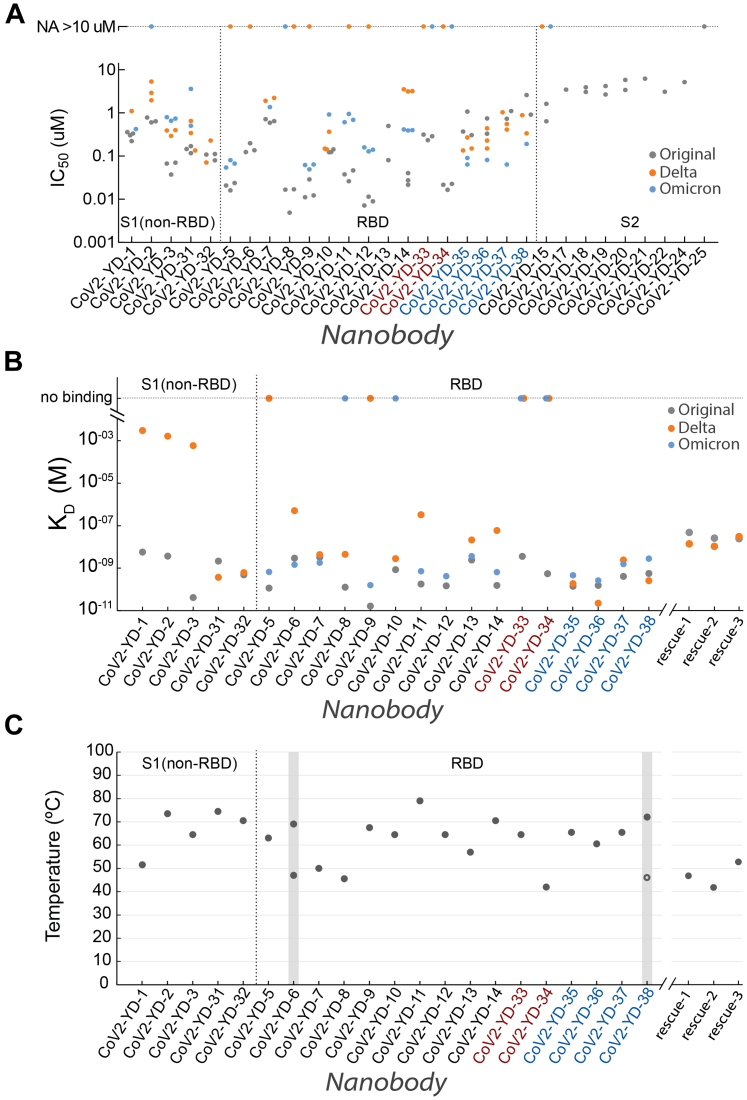
**Biophysical and neutralization properties of the nanobodies.** 30 yeast display nanobodies targeting the S1-RBD, S1 non-RBD, and S2 portions of spike were functionally tested for neutralization of lentivirus pseudotyped with various SARS-CoV spikes and their biophysical properties characterized. (*Red text* indicates YERLAWD family members, and *blue text* indicates NAAAW family members, used in the crossover “rescue” studies; see text). *A*, neutralization data against Original, Delta, and Omicron. *B*, Kd measurements of 21 of these nanobodies were determined using SPR and affinities plotted. Nanobodies were tested against Original, Delta, and Omicron recombinant S1 or RBD. S1 non-RBD nanobodies were not tested against Omicron. Kd measurements for three of the “rescue” constructs plotted at *right*. *C*, the Tm measurements of the nanobodies in (*B*) were determined using DSF and plotted. CoV2-YD-6 and CoV2-YD-38 (highlighted in *gray*) resulted in two distinct melting peaks. *Open circles* indicate less proportion of this species in the sample. Tm measurements for three of the “rescue” constructs plotted at *right*. DSF, Differential Scanning Fluorimetry; RBD, receptor-binding domain.

**Fig S4:**
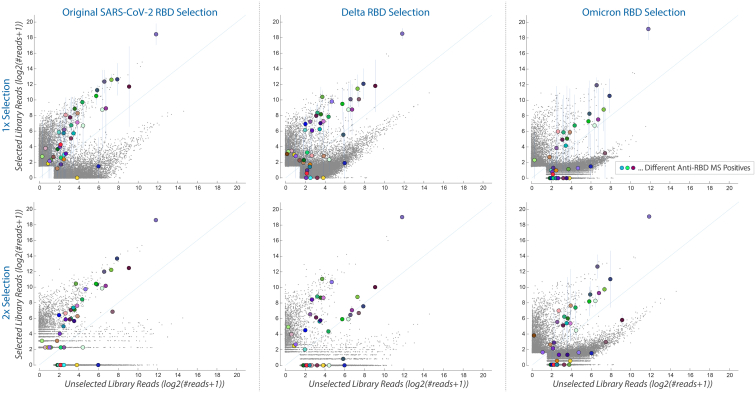
Testing CDR families of mass-spectrometry positives against RBD variants, Delta and Omicron. The data are plotted as in Figure 7 by plotting CDR families of the mass spectrometry-positive clones; each different color of the dots plotted represents a different mass spectrometry-positive nanobody.

One of the greatest challenges to managing COVID-19 is the ability of the SARS-CoV-2 virus to mutate into new VoCs that can resist prevalent vaccines and therapeutics. An advantage of generating large repertoires of nanobodies is that one maximizes the likelihood of finding VoC-resistant, broadly specific nanobodies (20, 47, 48, 49). At the time of writing, two variants and derivatives thereof remain of significant concern, namely Delta and Omicron. We wanted to evaluate the utility of yeast display for analyzing the sensitivity of our nanobody repertoire to these variants, focusing on the RBD domain as it is the region of largest mutational variation (50, 51) and the 7704 animal as it has the largest representation. We conjugated recombinant Delta and Omicron RBD to Dynabeads and tested for binding to the yeast library compared to the original SARS-CoV-2 strain’s RBD (two rounds of selection, with sequence analysis after each). Remarkably, overall, we observed that most sequences bound well to both variants (Figs. 6*A* and 7). For example, the large “TVDAQSDY” (Fig. 5*C*, (V)) family binds comparably to the original SARS-CoV-2 and both variants. The “LRSRFNAAAWTTEAAFDY” (“NAAAW”) (Fig. 5*C*, (iv)) family binds comparably to the original SARS-CoV-2 and the Delta VoC RBDs and appears collectively slightly weaker against the Omicron RBD. The “YERLAWDTSTY” (“YERLAWD”) (Fig. 5*C*, (iii)) family binds well to the original SARS-CoV-2 but binding is essentially eliminated for both variants. The “IIDDYGVQY” (“IIDDY”) (Fig. 5*C*, (vi)) family (Fig. 6*A*, “IIDDY”) and a family not indicated on Figure 5 but comprising a family characterized by a “TADLYSDY” (“TADLY”) CDR3 sequence binds well to original SARS-CoV-2 and the Omicron variant but more weakly to the Delta variant, especially after two rounds of selection. These families contain considerable sequence diversity within them (Fig. 5). There are many other unrelated clones (often with lower representation in the library) that exhibit similar behaviors (Fig. 6*A*), greatly expanding the useful repertoire. A similar pattern of behavior was seen for the “MS positives” in our screening (Fig. S4). This screening method is therefore potentially a rapid and straightforward way to further characterize the library for the VoC-specific sensitivities of the positive clones.

### Testing the modularity of CDRs by DNA shuffling

DNA shuffling is an established *in vitro* method for improvement of binding or catalytic activity and has previously been applied to nanobodies with recombination between CDRs, resulting in significant improvements of binding noted in the progeny (52, 53, 54). Typically it is applied to a library of randomly point-mutagenized sequences that have been selected for improved activity, from which starting point splice-overlap-extension (SOE) PCR is carried out to produce mix-and-match recombinants. Two advantages ensue from this method compared to simple clonal descent by cycles of mutagenesis and selection. First, deleterious mutations hitchhiking with selected mutations are readily crossed away; second, combinations of positive mutations in different regions can be combined in a single jump through sequence space that might be highly unlikely to occur by single-mutation steps. Improved activity and even new biological activities entirely lacking in the starting material can potentially be found in the products. It is interesting that while point mutagenesis is a biological strategy naturally used in the process of somatic hypermutation to improve antibody affinity (see Introduction), DNA shuffling is much rarer in natural biological systems. VDJ recombination shares some features, but with the critical difference that only a single round of shuffling occurs rather than multiple rounds interleaved with the recombinations. Neither the extremely high density of recombination joins that can be attained by *in vitro* shuffling nor its highly multiparental nature are shared by natural biological systems, to our knowledge.

**Fig S5:**
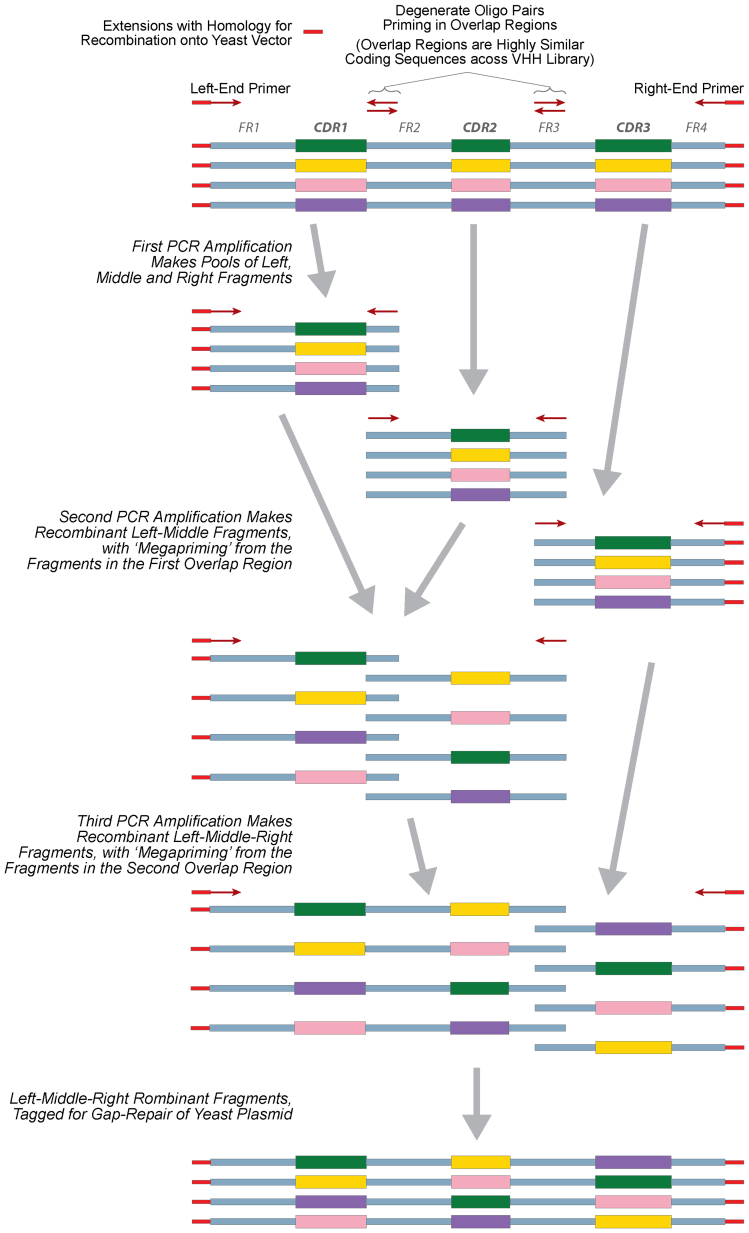
Diagram of the splice-overlap extension method for recombining CDRs. Degenerate oligos priming in both directions from two highly conserved sequences within framework regions (FRs) 2 and 3, and end oligos tagged for recombination onto the yeast expression vector, were used to amplify fragments containing CDRs 1, 2, and 3 as well as flanking framework regions. The template used was a pool after one round of selection on RBD. The result of the sequential PCR reactions indicated is a random mix-and-match of the three CDRs and flanking framework regions.

We started with the library selected on SARS-CoV-2 RBD and carried out SOE recombining CDRs 1, 2, and 3 at random from that library (Fig. S5). The advantage of this approach is that the specificity of the large number of input positive sequences is known, unlike in previous approaches (52, 53, 54). We bound the recombinant library (containing about 10^6^ members) to RBD-Dynabeads, and carried out next-generation sequencing on both the input shuffled library and the selected sequences. This allowed us to quantitatively evaluate the degree of enrichment over the shuffled, unselected library.

We expected that the incompatibility of CDRs from entirely unrelated nanobodies would result in a large majority of the shuffled progeny having highly reduced binding activity; however, this was not observed. A high proportion of the shuffled library could bind to RBD-Dynabeads at least to some extent. We organized the data by first examining the fate of specific CDR3 sequences, and then examining what CDR1 and CDR2 sequences were associated with high or low binding to RBD (either the original sequence (henceforth here termed Original) or the Delta and Omicron variants, as described already for the initial library (Fig. 6*A*)). In general, different CDR3 groups retained similar overall specificity of binding to Delta and Omicron to what was seen in the corresponding native families (Fig. 6). However, a notable exception was observed with the “YERLAWD” CDR3. The native YERLAWD family bound efficiently to Original but not to Delta or Omicron RBD. The shuffled YERLAWD group, in contrast, contained abundant members that bound equally well to Original and Delta RBD, while remaining almost completely defective in Omicron binding. Examination of the sequences associated with this high Delta binding revealed specific enrichment of sequences highly similar to the native “NAAAW” CDR1 and CDR2, recombined with the “YERLAWD” CDR3 (Fig. 6*B*). One explanation of this observation could be that the native “NAAAW” family’s CDR1 and CDR2 have a specific ability to bind to Delta RBD, independent of CDR3 content. However, direct examination of all shuffled products containing these CDR1,2 sequences shows that many fail to bind Delta RBD; binding requires specific CDR3s as well. So, fusion of CDR1,2 from the native NAAAW family to the YERLAWD CDR3 may create a fusion with effective multipoint attachment to the Delta RBD.

We biochemically characterized this apparent “rescue” of ‘YERLAWD” binding to Delta RBD by expressing four distinct “NAAAW” native parents and two “YERLAWD” native parents and recombinants between them as monomeric recombinant nanobodies (Fig. 7*B*; see also next section). As expected from the yeast display data (Fig. 6), the two “YERLAWD” parent nanobodies failed to bind to Delta RBD, while binding strongly to the Original RBD, and the four “NAAAW” parent nanobodies bound strongly to RBD from both strains (Fig. 7*B*). However, the hybrid nanobodies bound less well to Original and Delta RBD than either of their parents (with approximately equal affinities to Original and Delta), failed to neutralize (not shown), and showed denaturation at relatively low temperatures (Fig. 7*C*). These defects could simply reflect minor folding incompatibilities in the shuffled construct. In any case, the shuffling results overall suggest significant modularity in CDR function and could provide a novel avenue to new specificities which could be useful in diverse contexts.

### Neutralization and biophysical characterization of the isolated nanobodies on VoCs

We selected 30 abundant sequences that represented a cross-section of the major families (Fig. 5 and Table S1), most of which were distinct from clones previously isolated (20). We used the bacterially expressed and purified monomers to determine binding affinities and neutralization capability, using assays we have employed previously (20); we also tested binding affinities and neutralization against Delta and Omicron variants (Fig. 7).

With regards to neutralization, of the 21 S1-targeting nanobodies tested (both RBD and non-RBD binding), all 21 were capable of neutralizing Original, while 10 were still able to neutralize both Delta and Omicron, although with reduced activity against the variants in some cases. We also tested nine anti-S2 nanobodies for neutralization of Original; some neutralization activity was observed, although less efficient than for the better of the anti-S1 nanobodies. Such reduced neutralization was also true of our previously characterized anti-S2 nanobodies (20). A subset of the anti-S1 nanobodies were tested for binding affinity against recombinant S1 or RBD from either Original, Delta, or Omicron (S1 non-RBD nanobodies were not tested against Omicron) (Fig. 7*B*); all bound strongly to Original, displaying affinities in the nM to pM range. Two of these failed to bind only Delta, two failed to bind only Omicron, and CoV2-YD-33 and CoV2-YD-34 failed to bind both variants. Aside from CoV2-YD-10, all were in agreement with their failure to neutralize Delta, Omicron, or both strains, and some had a moderate to strongly reduced binding to Delta and Omicron, again correlating approximately with their reduced neutralization for that strain (Fig. 7*A*). CoV2-YD-10 neutralizes yet shows no binding to the RBD of Omicron using surface plasmon resonance (SPR). It is possible the binding site of the nanobody may be slightly truncated in the RBD construct used for SPR, resulting in the no-binding result. Lastly, all showed a moderate to strong degree of thermal stability, typical of nanobodies (Fig. 7*C*) (21).

Overall, the nanobody binding in the yeast display screening (Fig. 6) correlates reasonably well with that seen in the biochemical assay of the corresponding expressed nanobody (Fig. 7*B*). Thus, the differential affinities of the “YERLAWD” and “NAAAW” nanobodies for Delta RBD in both yeast display screening (Fig. 6) and the biochemical assay of the correspondingly expressed nanobody (Fig. 7*B*) agree, as discussed in the previous section. “LAYVT” (CoV2-YD-7) shows no significant loss of affinity for either Delta or Omicron RBD in both yeast display screening and in the biochemical assay of the correspondingly expressed nanobody. “TALLS” (CoV2-YD-6) show partial loss of affinity for Delta RBD while retaining Omicron affinity in both yeast display screening and in the biochemical assay of the corresponding expressed nanobody. “TADLY” (CoV-YD-9) shows complete loss of affinity for Delta RBD in both yeast display screening and in the biochemical assay of the corresponding expressed nanobody, while again retaining Omicron affinity in both assays. One nanobody seems something of an exception: “IIDDY” (CoV2-YD-8) has lost binding for Omicron RBD in the biochemical assay but no obvious reduction in the yeast display screening; perhaps the avidity effect of the display method compensates for the loss of affinity of the monomeric nanobody (20). For the bulk of the new nanobodies tested, the magnitudes of their binding affinities, neutralization potentials, and thermal stabilities were comparable to those of the nanobodies characterized in (20). Collectively, these similar patterns of behaviors of the selected representatives of this new yeast display repertoire to that of the previously published MS-identified repertoire (20) strongly suggest that the same modifications found in that work to powerfully enhance affinity or neutralization activity – multimerization and synergistic mixtures – will be equally applicable to this current repertoire.

### Epitope mapping of the nanobody repertoire

**Fig S6:**
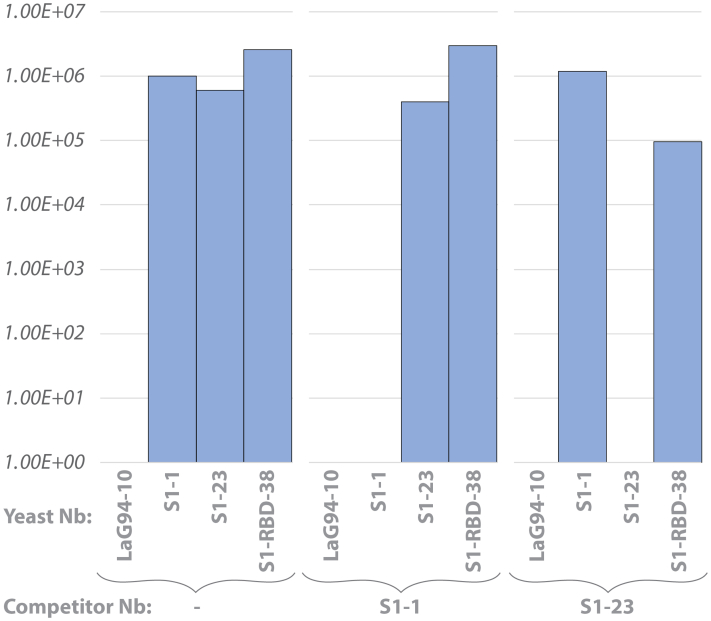
Epitope binning tests. Dynabeads conjugated with RBD were preblocked with saturating amounts of either monomer nanobodies S1-1 or S1-23, with unblocked Dynabeads as control. Yeast expressing the anti-RBD nanobodies S1-1, S1-23, or S1-RBD-38 (each belonging to different epitope classes as defined by (20)), or the anti-GFP nanobody LaG94-10 were bound to these beads. After binding and washing, we quantified recovery of yeast by colony counts of serial dilutions on yeast medium. S1-1-expressing and S1-23-expressing yeast were blocked from binding by the cognate but not the non-cognate nanobody block.

Another important characterization of any nanobody repertoire is to determine the different epitopes being recognized by each nanobody, as in the case of anti–SARS-CoV-2 S nanobodies, exploration of a larger epitope space increases the likelihood of discovering variant-resistant, strongly neutralizing nanobodies (20). This is usually done by “epitope binning”: finding classes of nanobodies that reciprocally inhibit each other’s binding due to competition for the same epitope. Epitope binning is generally carried out by one-on-one competitions between pairs of nanobodies; therefore, the number of assays scales with the square of the number of nanobodies to test. It occurred to us that we could use the yeast display assay to determine nanobody sequences in a given epitope bin across the entire library in a single binding experiment. We tested this idea in a preliminary experiment in which we saturated RBD-Dynabeads with three different nanobodies, each previously shown to bind to a distinct epitope (20). We then tested those beads for binding to yeast cultures, each expressing a single nanobody in a known biochemically defined epitope bin. Prior nanobody binding quantitatively blocked binding of yeast-expressing nanobodies in the same epitope bin but had essentially no effect on binding of yeast-expressing nanobodies known to be in different epitope bins (Fig. S6), thus validating the approach.

**Figure 8:**
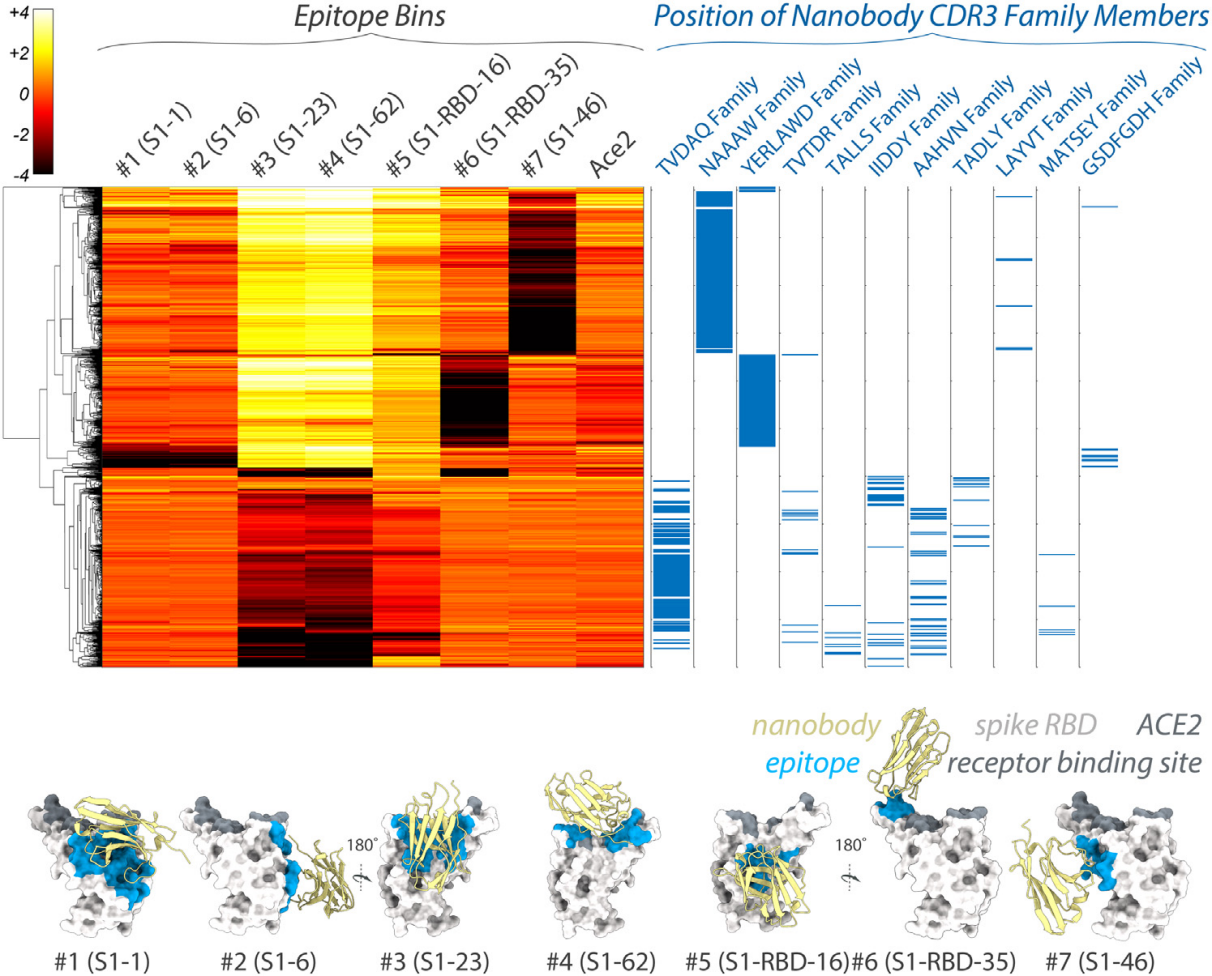
**Epitope binning by yeast display.** Dynabeads conjugated with receptor-binding domain (RBD) were blocked with monomer nanobodies representing the 7 epitope classes defined previously (20); with the soluble extracellular domain of the RBD target Ace2; or left unblocked. The 2× -RBD-selected library from llama 7704 (Figs. 4, 5 and S1) was bound to these beads. The bound VHHs were sequenced, and enrichment/depletion upon blocking for each “CDR string” (catenated CDR1/CDR2/CDR3) was calculated as log2(read count with blocked beads/read count with unblocked beads). The sequences were filtered to remove PCR crossover artifacts (Methods). *Left*: The resulting matrix of ∼100,000 sequences X 8 blocking agents was filtered for read count (at least 100 reads combining all blocking experiments) and clustered using the MATLAB hierarchical clustering algorithm; scale bar on left indicates log2 enrichment/depletion (above). *Right*: The location of sequences containing the indicated CDR3, or close relatives, was determined. Note that the hierarchical clustering was sequence-blind, so the clustering of related sequences implies similar blocking behavior across the family. *Bottom*: Representative nanobodies (*yellow ribbon*) binding to their respective epitopes (*blue surface patch*), as defined previously (20), are depicted on the RBD of SARS-CoV-2 spike (PDB ID: 6M0J). ACE2, angiotensin-converting enzyme 2.

To carry out parallel epitope binning for the entire yeast display library, we followed the conditions from this preliminary experiment: RBD-Dynabeads were saturated with seven different nanobodies, each determined to represent seven epitope classes using an integrative mapping approach which incorporated epitope binning by competitive binding, then subdivided the biochemical epitope bins based on escape mutant and crosslinking-MS data (20) (we will refer to these subdivided bins as “epitope classes”). As with biochemically defined epitope bins, the classes may overlap on the RBD surface. We also attempted to block with the soluble extracellular domain from ACE2. The blocked beads were used to select binders from a library of twice-selected RBD binders (Figs. 4 and 5). V_H_H sequences from the bound population were determined, and the read counts of the sequences bound to RBD beads blocked with each nanobody were determined. The read count recovered from the blocked beads was divided by the read count from the unblocked beads, and the resulting ratios hierarchically clustered (Fig. 8). These seven epitope classes were selected to collectively encompass essentially all of the available RBD surface (20). Consistent with this, the majority of nanobodies in our population are inhibited by blocking the RBD beads with at least one of the seven nanobody classes (Fig. 8). A minority of nanobodies were not so inhibited and may represent new epitope class(es).

Many nanobodies fall into more than one epitope class, as defined by this assay. Thus, most nanobodies in class #1 are also in class #2, and vice versa, and a similar mutuality is seen between the classes #3 and #4, which in turn contains a smaller subgroup that is also found in class #5 (Fig. 8). The positions of the “founding” epitopes for these classes were estimated previously from MS cross-linking data and escape mutants (20). Examination of the estimated position of these epitopes on RBD indeed indicates that there is significant overlap or adjacency between #1 and #2 and between #3 and #4 (and even #5 or #6), consistent with steric clashes that could lead to the class overlaps observed (Fig. 8) (20).

We checked the fidelity of this method by examining behaviors of nanobodies previously identified and classified (20). In total, that work placed 17 nanobodies in one of these seven epitope classes (20). Of those 17, 11 were found in the library; of those 11, 9 exhibited the expected result of specific prevention of binding to RBD beads blocked with the nanobody defining the epitope class. The remaining two were less clear, with a related sequence found for one (S1-RBD-21) showing some inhibition in epitope class #6 though previously assigned to #4 and the other (S1-RBD-23) showing no inhibition though previously assigned to class #5.

**Fig S7:**
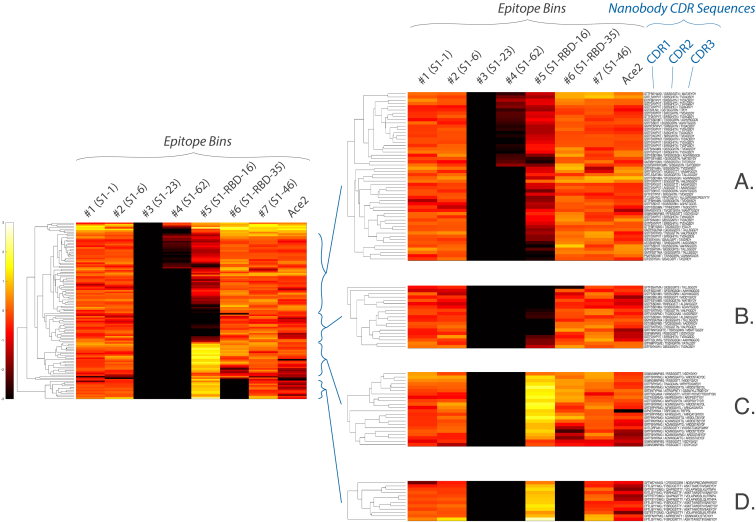
Epitope Subclusters of families. Left: same as Figure 8 left, for reference. The indicated subclusters were extracted using the MATLAB clustergram tool and CDR sequences recovered (*right*).

**Fig S8:**
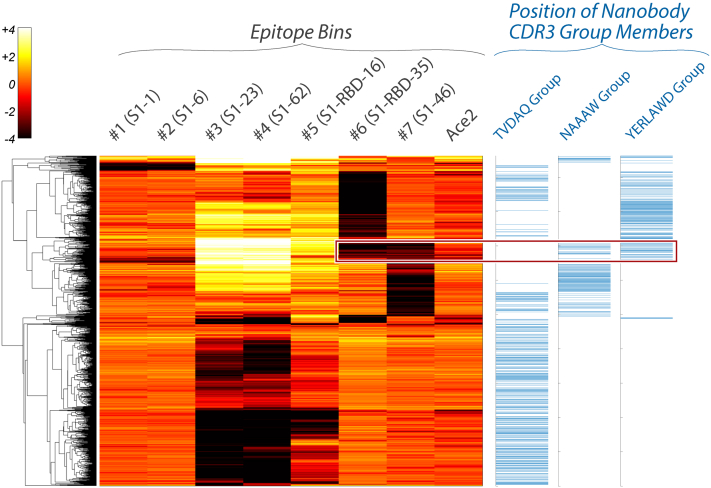
Crossover epitope binning. The sequences clustered in Fig. 8 were curated computationally to eliminate a population of PCR crossover events (see Methods) these are essentially similar to the ‘shuffled’ products made intentionally (Fig. 6). We analyzed these crossover products separately (*left*) and observed intriguing behavior in some cases. For example, while blockage by #6 and #7 was mutually exclusive in the native sequences (Fig. 8), crossover products with CDR1 and 2 derived from the “YERLAWD” native sequence and CDR3 from the “NAAAW” native family were blocked by *both* #6 and #7; the converse recombinants (CDR1/2 from “NAAAW” and CDR3 from “YERLAWD”) behaved similarly (these sequences provide almost the entire signal in the boxed region in the figure). Numerous other examples of similar apparent “hybrid” epitope classes were observed among the crossover recombinants. Biochemical analysis would be required to determine if such behavior represents a bona fide two-part epitope with CDR1/2 binding at one place and CDR3 binding at another; however, at present we cannot exclude that the “hybrid epitope classes” are specific to the potentially multipoint attachment of polyvalent yeast to a polyvalent binding surface.

We further dissected nanobody binding behaviors in this assay by once again following particular nanobody families as defined by their CDRs (as above; Figs. 5 and 6). The sequence variants within families generally fell together on the clustergram, which was generated sequence-blind, based solely on the binding behavior in the seven blocked populations. This result supports the similar behavior of almost all the sequence variants assigned to the families. For example, essentially all of the sequences in the “YERLAWD” CDR3 family were specifically blocked by #6, and essentially all of the sequences in the “NAAAW” and “LAYVT” CDR3 families (Figs. 5 and 6) were specifically blocked by #7 (Fig. 8). Many other smaller CDR3 families also displayed similar behaviors. Interestingly, classes #1 to #4 did not singly block any significant CDR3 families but rather acted to block them in different combinations. Thus, many of the CDR3 families were blocked by both #3 and #4, of note being the large CDR3 family characterized by the starting sequence TVDAQ; however, there appears to be a range of behaviors in this large class (Figs. 8 and S7). So, some CDR3 families are essentially exclusively blocked by #3 and #4, such as those CDR3 sequences characterized by CDR3s starting with ARDD, ARNQ, and WRYF (Fig. S7, group C). A number of families are similarly strongly blocked by #3 and #4 but also partially blocked (to lesser or greater extents) by #5 (Figs. 8 and S7, group A); these include the large TVDAQ, TALLS, and AAHVN CDR3 starting sequence families (Fig. 8; see also Figs. 5 and 6). Then there are those nanobody CDR3 families almost equally strongly blocked by #3, #4, and #5, including S1-RBD-43 and related nanobodies (characterized by the CDR3 starting sequence AGHV) (Fig. S7, group B). Lastly, there are nanobodies that are blocked relatively equally by classes #3, #4, and #6, notably those with CDR3 sequences starting with VDLAP or ASKTT (Figs. 8 and S7; group D). Thus, the epitope classifications are not absolute; there are varying degrees of relative inhibition for each nanobody compared with its neighbors on the plot that indicate there are a very large number of discrete, though frequently overlapping epitopes recognized by the population. In contrast, epitope classes that were deduced to be far apart on the RBD surface, such as #6 and #7, exhibit little or no overlap of depletion (intriguingly, analysis of crossover recombinants breaks this rule (Fig. S8) for unknown reasons). Within these data are results that also serve to highlight new families not discussed above, some with similar epitope class behaviors as the more common families, and also many that show new behaviors - and among them are also MS-identified with previously unknown specificity, such as close relatives of S1-RBD-16, which is blocked by both #5 and #7 epitope classes. These behaviors suggest that the population of nanobodies effectively “scans” the available epitope space.

## Conclusions

We show that the version of the yeast display method presented here is capable of generating a large repertoire of high-affinity nanobodies. A marked advantage of the current method is its robustness, simplicity, and relatively low cost in time and resources; it avoids the need for complex and expensive instrumentation and the associated necessary expertise. We contend that any laboratory with standard resources and skill sets can readily adapt this method to generate nanobodies against various targets; commercial camelid and sequencing resources can easily be remotely accessed, as they were for this work.

We also show three further adaptations of this method. The first is its use in combination with our previous biochemical/MS methods (20, 22). We show that in this way, one can significantly increase the total nanobody repertoire. Alternatively, we propose that one can adopt a “two factor” identification approach – only selecting nanobodies that are positive in both the biochemical/MS and yeast display methods, these being virtually certain to be true positives with high affinities. Large repertoires are extremely advantageous to maximize epitope space, affinity, the likelihood of obtaining a desired biological activity such as viral neutralization, and candidates for advantageous synergistic mixture or oligomers of nanobodies. The second is the ability to shuffle between different versions of each CDR, which may, in certain cases generate nanobodies with new binding behaviors and allow exploration of CDR modularity across the library. The third is that the method can be adapted to allow massively parallel epitope binning. Epitope binning is conventionally and painstakingly performed one nanobody pair at a time. Our method for parallelized epitope binning, in contrast, allows testing many thousands of candidates at once against each known nanobody epitope. This is a necessary step on the road to generating fully characterized nanobodies, including those with diagnostic or therapeutic potential. Indeed, here we have generated many new nanobodies with strong neutralization activity that may be further adapted into treatments for the continuing fight against COVID-19 (20).

## Experimental procedures

### Library construction

Starting with B cell cDNA from the same immunized llamas described previously (20), we amplified V_H_H sequences with oligos providing flanking homology for cloning into the yeast display vector (34). We carried out gap-repair using a high-efficiency yeast transformation method (55), which in our hands yielded a maximum efficiency of colony recovery of ∼1.5 ∗ 10ˆ7 colonies. We used a diploid trp1- W303 strain as recipient, selecting on ScMin-2% glucose. We experienced sporadic culture contamination problems with environmental fungi; we found that use of canavanine (ScMin+can+lys) controlled this problem to a manageable level due to the *can1* canavanine-resistance mutation in W303. For control experiments, we subcloned anti-GFP Nbs (22), also the anti-RBD nanobodies S1-1 and S1-23 (20), using standard cloning methods.

### Surface nanobody induction

Stationary phase yeast were diluted 1:5 into 0.2% glucose-6%galactose in Sc-Min, grown overnight at 30 °C with rotation, supplemented with 10% volume fresh Sc-Min and an additional 3% galactose for another 24 h. These conditions gave strong induction of displayed nanobodies as detected using anti-GFP controls included in all experiments, so that we could detect surface nanobody by labeling cells with purified GFP. As described previously (56) we noted a substantial population of cells negative for GFP binding; plating experiments strongly suggested that these were due to plasmid loss events somehow induced by galactose incubation. Since these cells were plasmid-free they did not contribute to any downstream steps (outgrowth in selective medium or subsequent amplification of plasmid sequences) and therefore were inconsequential.

### Yeast affinity capture

All antigens were conjugated to Thermo Fisher Scientific Dynabeads (M-270 epoxy 14,301), following the manufacturer’s protocol with minor adaptations (57, 58); GFP was made in-house (22), and the appropriate His-tagged SARS-CoV-2 S antigens were obtained from Sino Biologicals Inc: S RBD, S1, or S2 ECD (original strain); S RBD L452, T478K (Delta); and B.1.1.529 (Omicron) S RBD. Yeast were vitally fluorescently labeled on their cell surfaces as previously described (40). We used GFP-Dynabeads and yeast expressing surface anti-GFP to establish conditions for binding and washing. The optimal binding buffer we discovered is described below. A 1 h binding of yeast to beads with rotation at 30 °C was followed by 4 to 5 washes with purification of bead-bound cells on a magnet using a Dynal MPC-6 magnetic stand, with samples kept at 2 cm from the magnet, 5 min binding per wash. All yeast affinity captures were performed in 1% BSA (Fraction V, protease-free; GoldBio (St Louis, MO)), 1× PBS (137 mM NaCl, 2.7 mM KCl, 10 mM Na_2_HPO_4_, 1.8 mM KH_2_PO_4_ pH to 7.4 with HCl/NaOH), 1% Tween-20 (Sigma Aldrich). For the epitope binning, affinity capture, and subsequent library generation of the yeast was performed as described above except that each 10 μl aliquot of the RBD-conjugated Dynabeads were preblocked with the addition of 20 μg of the appropriate nanobody or Ace2 in 1% BSA, 1 × PBS, 0.1% Tween-20 rotating for 1 h at room temperature. Affinity capture with Miltenyi beads and subsequent FACS were performed as described (34).

### Sequencing of nanobody clones in the purified yeast library

After binding, beads with bound cells were transferred to ScMin-2% glucose and grown out for 14 to 48 h. Cells were pelleted, lysed with Zymolyase and DNA purified on Qiagen miniprep columns following manufacturer’s procedures. The DNA prep was amplified with sequencing primers and sequenced at the Rockefeller Genomics facility using an Illumina MiSeq, PE250 (early experiments), and PE300 (most experiments; better sequence quality due to longer overlap between the paired reads).

### Nanobody cloning, expression, and characterization

Cloning, expression, and purification of the nanobodies, SPR, Differential Scanning Fluorimetry and SARS-CoV-2 Pseudovirus Neutralization Assays were performed as described (20).

### Computational methods

Nanobody sequences were obtained by paired-end sequencing (300 bp read length) using Illumina MiSeq. Since each nanobody sequence was potentially represented by exactly one pair of reads, it was important to filter the data for quality. The computation was as follows: for positions covered only by one of the two paired-end reads, the quality score for that position was the one assigned by MiSeq. For positions covered by both of the paired-end reads (*i.e.*, both strands sequenced), the base call was that for the higher quality-scored position, and the final score was the sum of quality scores for the two reads if the base call was the same, and the higher minus the lower score if the base call was different. The overall nominal probability of having no error anywhere in the sequence was then computed as 1 – Product(-Q/10), product taken over all positions in the sequence, where Q is the final quality score at each position, and a cutoff of 0.9 applied. In addition, a similar calculation was applied to sequences approximately encoding CDR1, CDR2, and CDR3, with a cutoff of 0.95.

The CDR sequences were extracted from the complete nanobody sequence using the consensus FR region sequences from (41). Their consensus sequences were used to generate multiple alternate FR regions (usually with one or two substitutions each) and the best alignment to each FR (testing separately all of the candidate FR regions) was found. Sequences between FRs were assigned as CDRs 1,2, and 3. Subsequent computations were done using the “CDR string” composed of the catenated CDR1,2, and 3 sequences. Due to minor FR variability, there were approximately 1/3 as many CDR strings as full nanobody sequences. The data indicated strong concordance among nanobodies with the same CDR string, consistent with the known primacy of CDR sequence for binding specificity (see Introduction).

For all libraries, the number of sequences (nanobody or CDR string) was standardized to the total size of the library. Read count was then adjusted to reads per million. The result was a table with rows corresponding to sequence and columns to standardized read count in a series of libraries. We created a custom viewer to compare read count in various categories (*e.g.*, in specified polygons in a 2-D graph; containing some CDR3 sequence; etc). The viewer employed log2(standardized read count + 1); thus zero reads were plotted at 0, 1 read at 1 and higher read counts plotted at approximately log2(read count).

Crossover sequences (almost surely derived by PCR template switching within the highly homologous FR regions) were identified as follows. Sequences (CDR strings) were ranked in the order of abundance. The most abundant initiated a list of “native” (noncrossover sequences). Subsequent (decreasing abundance) sequences were then examined for a good match in some CDRs to a sequence in the “native” list combined with a bad match in other CDRs. Such cases were assigned to a list of “crossover” sequences; others (either distinct in all three CDRs or similar in all three CDRs to members of the native list) were appended to the native list.

All computations were carried out by MATLAB code, available upon request. Sequence logos and phylogenetic trees were calculated using built-in functions in the MATLAB Bioinformatics toolbox.

For the blocking experiment, we used the standardized read counts to calculate depletion/enrichment based on #reads in blocked library/#reads in unblocked library. This was done after filtering out likely crossover products (see above).

## Data availability

All data generated or analyzed during this study are included in this published article (and its supplementary information), or are available from the corresponding authors on request.

## Conflict of interest

The authors declare that they have no conflicts of interest with the contents of this article.
